# Distinct modulation of IFNγ-induced transcription by BET bromodomain and catalytic P300/CBP inhibition in breast cancer

**DOI:** 10.1186/s13148-022-01316-5

**Published:** 2022-07-28

**Authors:** Simon J. Hogg, Olga Motorna, Conor J. Kearney, Emily B. Derrick, Imran G. House, Izabela Todorovski, Madison J. Kelly, Magnus Zethoven, Kenneth D. Bromberg, Albert Lai, Paul A. Beavis, Jake Shortt, Ricky W. Johnstone, Stephin J. Vervoort

**Affiliations:** 1 Gene Regulation Laboratory, Peter MacCallum Cancer Center, 305 Grattan Street, Melbourne, VIC 3000 Australia; 2 Sir Peter MacCallum Department of Oncology, The University of Melbourne, Melbourne, Australia; 3 Monash Haematology, Monash Health, Clayton, Australia; 4 Cancer Immunology Program, Peter MacCallum Cancer Center, Melbourne, Australia; 5 Oncology Discovery, AbbVie, North Chicago, IL USA; 6 School of Clinical Sciences at Monash Health, Monash University, Clayton, Australia; 7 Present Address: Oncology Discovery, AbbVie, South San Francisco, CA USA; 8 Present Address: The Walter and Eliza Hall Institute of Medical Research, 1G Royal Parade, Parkville, VIC 3052 Australia

**Keywords:** Interferon, Histone acetylation, Bromodomain, P300/CBP, H3k27ac, Inflammation, Enhancer, Immuno-oncology

## Abstract

**Background:**

Interferon gamma (IFNγ) is a pro-inflammatory cytokine that directly activates the JAK/STAT pathway. However, the temporal dynamics of chromatin remodeling and transcriptional activation initiated by IFNγ have not been systematically profiled in an unbiased manner. Herein, we integrated transcriptomic and epigenomic profiling to characterize the acute epigenetic changes induced by IFNγ stimulation in a murine breast cancer model.

**Results:**

We identified de novo activation of *cis*-regulatory elements bound by Irf1 that were characterized by increased chromatin accessibility, differential usage of pro-inflammatory enhancers, and downstream recruitment of BET proteins and RNA polymerase II. To functionally validate this hierarchical model of IFNγ-driven transcription, we applied selective antagonists of histone acetyltransferases P300/CBP or acetyl-lysine readers of the BET family. This highlighted that histone acetylation is an antecedent event in IFNγ-driven transcription, whereby targeting of P300/CBP acetyltransferase activity but not BET inhibition could curtail the epigenetic remodeling induced by IFNγ through suppression of Irf1 transactivation.

**Conclusions:**

These data highlight the ability for epigenetic therapies to reprogram pro-inflammatory gene expression, which may have therapeutic implications for anti-tumor immunity and inflammatory diseases.

**Graphical Abstract:**

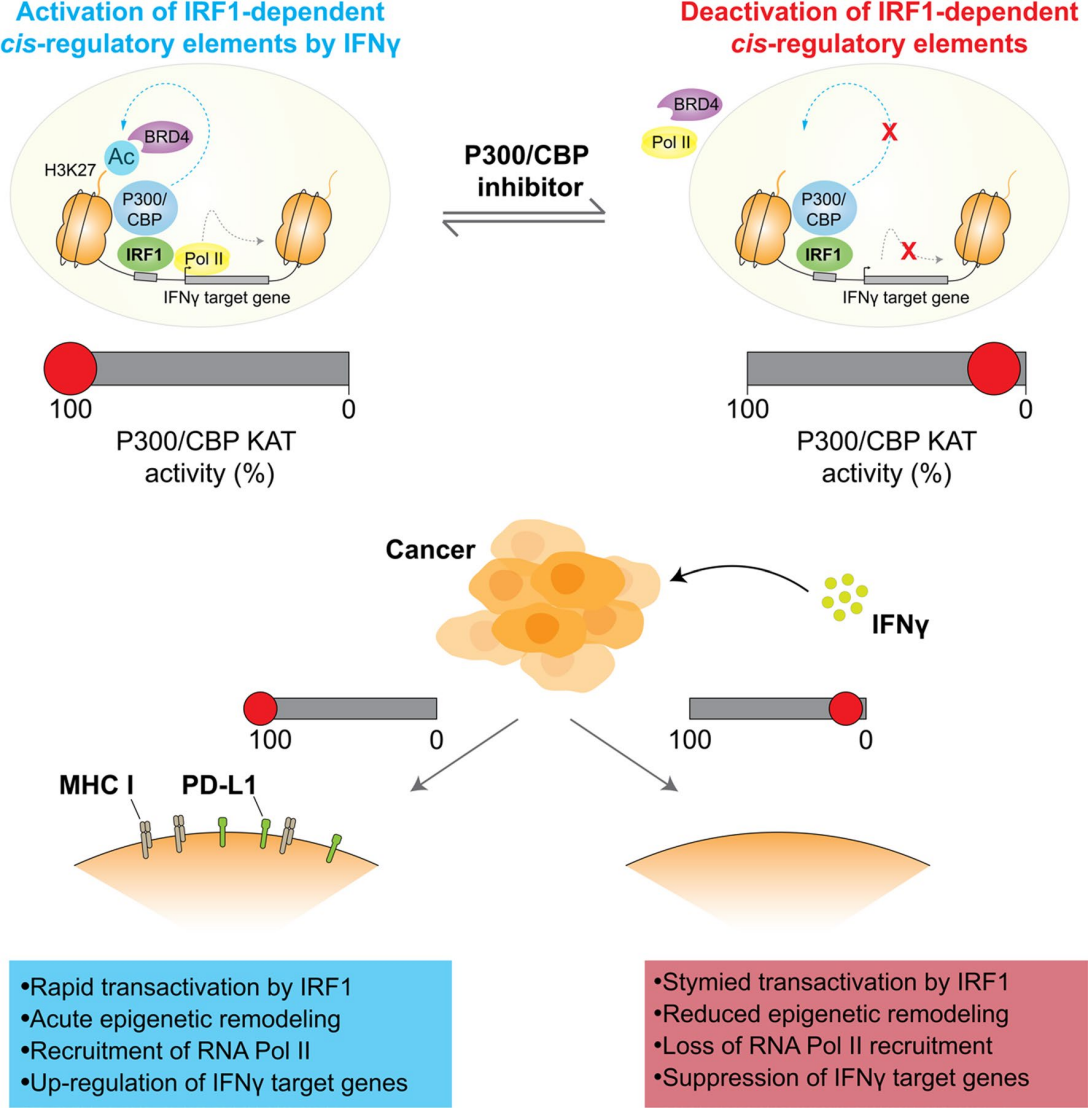

**Supplementary Information:**

The online version contains supplementary material available at 10.1186/s13148-022-01316-5.

## Background

Pro-inflammatory cytokines directly activate signaling cascades, however, epigenetic mechanisms are also critical for coordinated inflammatory gene expression. Interferon gamma (IFNγ) is a pleiotropic cytokine that is a key regulator of anti-tumor immunity [1, 2]. In the tumor microenvironment (TME), IFNγ induces the expression of genes essential for antigen processing and presentation, thereby promoting immune-surveillance [3]. Gene expression profiling in melanoma patients treated with nivolumab (anti-PD1) alone or combined with Ipilimumab (anti-CTLA4) revealed that an IFNγ-driven gene signature was the most predictive feature for clinical responses to these immunotherapies [4]. In contrast, genetic aberrations that suppress IFNγ signaling are detected in patients relapsing from immune checkpoint blockade demonstrating that tumor-intrinsic IFNγ signaling is a clinically relevant mechanism of immune evasion [5]. This is also supported by genome-scale immune evasion screens that recurrently identify the IFNγ receptors (IFNGR1/IFNGR2) and obligate JAK/STAT members as the most important mediators of sensitivity to T-cell killing [6, 7]. Finally, in vitro models of acquired resistance to T-cell bispecific antibodies and CAR T-cells also converged on IFNγ signaling as a key mediator of immune evasion [8]. Thus, anti-tumor immunity and therapeutic responses to immune oncology agents are critically determined by tumor cell intrinsic IFNγ signaling.

It is now well established that epigenetic therapies may modulate the immunogenicity of tumor cells and inflammatory gene expression in cancer [9]. For example, we, and others, identified that the pre-clinical activity of prototypical BET Bromodomain inhibitor, JQ1, was dependent on an intact host immune system, which was mechanistically linked to suppression of immune checkpoint ligand PD-L1 on tumor cells [10, 11]. Suppression of PD-L1 by BET inhibition was evident in the context of 9p24.1-amplified Hodgkin lymphoma, where PD-L1 is constitutively expressed [12], as well as triple-negative breast cancer (TNBC) as a model of IFNγ-dependent expression. While IFNγ-inducible expression of PD-L1 was BET protein-dependent, the wider role of BRD4 or additional epigenetic regulators in regulating cellular responses to IFNγ remains poorly defined.

Herein, unbiased transcriptional and epigenomic profiling was employed to provide mechanistic insight into the hierarchy of events occurring at the chromatin interface following IFNγ stimulation leading to the coordinated transcription of IFNγ target genes. These epigenetic events underpinning IFNγ-induced gene expression were uncoupled by antagonizing distinct nodes using small molecule inhibitors of epigenetic modulators. Taken together, these data illustrate the acute epigenetic remodeling that drives the IFNγ-induced transcriptional response.

## Results

### IFNγ promotes rapid transcriptional activation in solid tumors

**Fig. 1:**
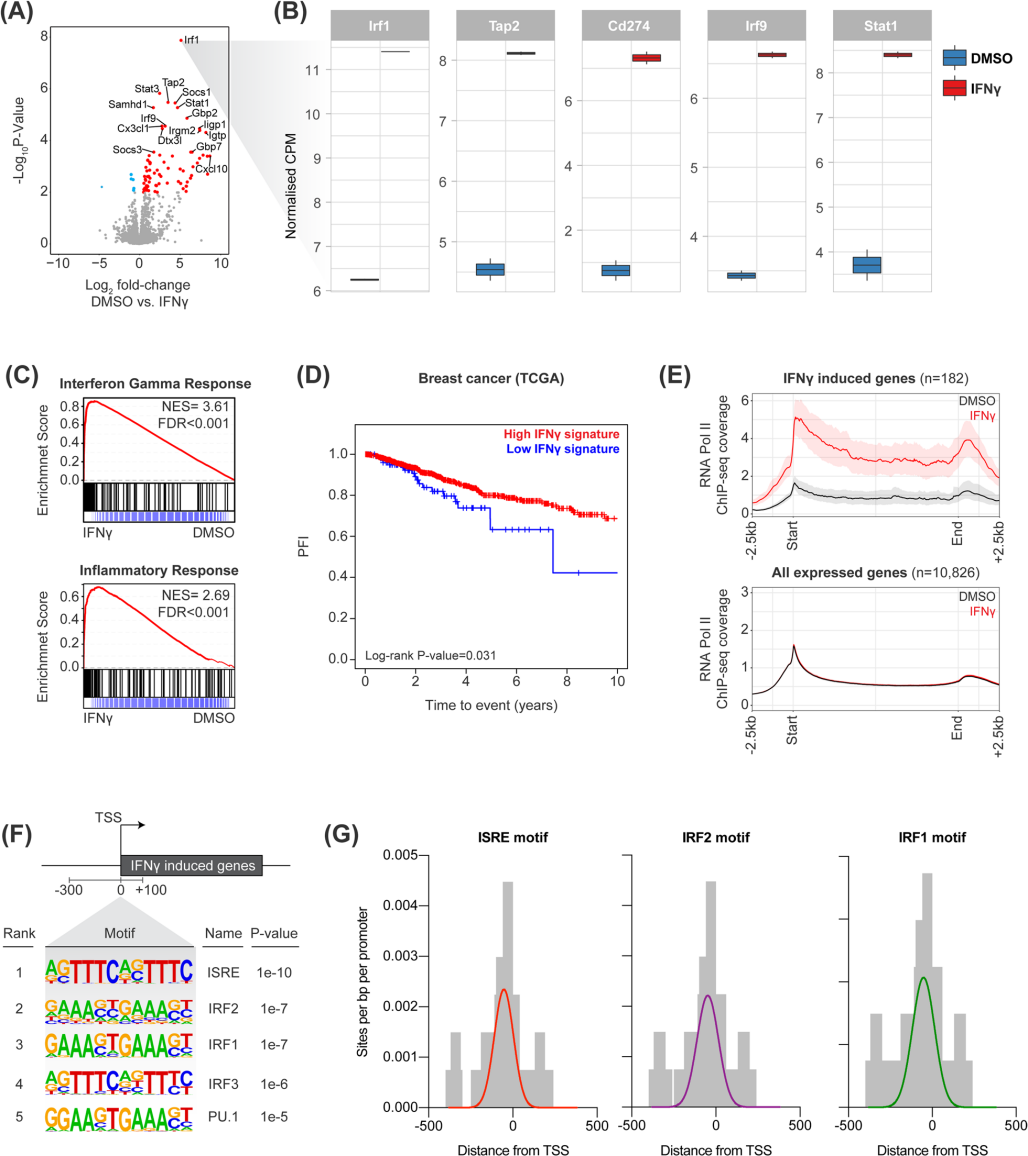
IFNγ evokes a concerted transcriptional response in cancer. **A** RNA-seq of AT3 cells stimulated with IFNγ. **B** Normalized counts (log_2_ counts per million) of IFNγ stimulated genes. **C** GSEA for AT3 cells stimulated with IFNγ. **D** Survival of breast cancer patients (from TCGA) stratified by expression of concordant IFNγ stimulated genes. **E** RNA polymerase II occupancy by ChIP-seq across IFNγ stimulated or non-IFNγ stimulated genes. **F** De novo motif analysis in promoters of IFNγ stimulated genes. **G** Distribution of IRF and ISRE motifs within promoters of IFNγ stimulated genes

We utilized the AT3 model of TNBC to profile the transcriptional and epigenetic effects of IFNγ. We first performed RNA-sequencing (RNA-seq) in AT3 cells treated acutely with recombinant IFNγ or vehicle control and subsequent differential gene expression analysis (DGEA) revealed that IFNγ stimulation led to a concerted transcriptional response (Fig. 1A) involving predominant up-regulation of numerous genes, including canonical IFNγ target genes *Cd274* (PD-L1), *Irf1*, *Tap1,* and *Socs1* (Fig. 1B). The transcriptional response in AT3 cells was also highly correlated with canonical IFNγ signaling and inflammatory gene expression by Gene Set Enrichment Analysis (GSEA; Fig. 1C). To identify the conservation of the IFNγ response, we analyzed RNA-seq of additional murine solid tumors treated with IFNγ, including B16F10 (melanoma) and MC38 (colon adenocarcinoma), which revealed concordant transcriptional responses (Additional file 1: Fig. S1A, B) across all cell types (Additional file 1: Fig. S1C). Importantly, the IFNγ signature had a strong prognostic significance in a TCGA cohort of breast cancer patients associated with high IFNγ signature positivity correlating with superior survival (Fig. 1D). These data indicate that the molecular processes that regulate tumor cell intrinsic IFNγ target gene expression are of high clinical relevance and prognostic significance in cancer.

Chromatin immunoprecipitation and sequencing (ChIP-seq) for RNA Polymerase II (RNAPII) was performed to assess the processivity across IFNγ-induced genes. IFNγ-stimulated genes were characterized by rapid de novo recruitment of RNAPII following IFNγ treatment with high levels of RNAPII occupancy across the coding region, concurrent with minimal promoter-proximal pausing (Fig. 1E). This indicates that unlike EGF responsive genes, which primarily rely on rapid release of RNAPII from the paused state [13], RNAPII initiation is a key rate-limiting checkpoint for IFNγ response genes. In contrast, non-IFNγ-regulated genes exhibited unaltered RNAPII occupancy (Fig. 1E). To gain insight into transcription factors (TFs) driving IFNγ-induced gene expression, de novo motif analysis was performed on the promoter regions of IFNγ-stimulated genes. This demonstrated significant overrepresentation of Interferon-Regulatory Factors (IRF) and the Interferon-Sensitive Response Element (ISRE) motifs (Fig. 1F, G). Overall, these studies indicate that acute IFNγ stimulation leads to active recruitment of RNAPII to a conserved subset of genes putatively regulated by IRF transcription factors.

### IFNγ stimulates acute epigenetic remodeling of IRF1-bound cis-regulatory elements

**Fig. 2:**
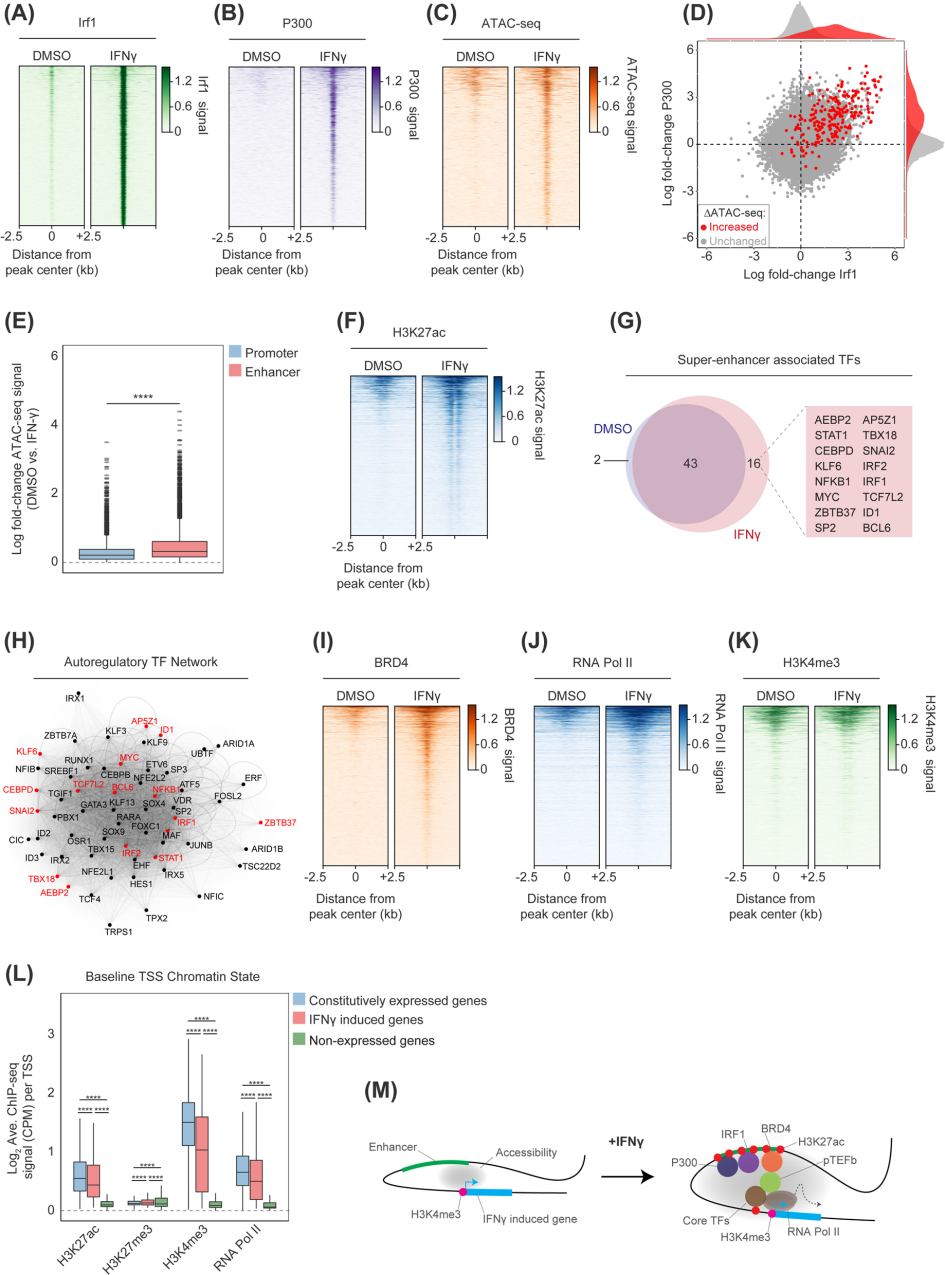
IRF1 binding drives de novo enhancer remodeling. **A** Binding of IRF1 by ChIP-seq to IFNγ-induced loci. **B** Binding of P300 by ChIP-seq to IFNγ-induced loci. **C** Chromatin accessibility by ATAC-seq at IFNγ-induced loci. **D** Correlation in IRF1 and P300 recruitment at sites demonstrating increased ATAC-seq signal. **E** Log_2_ fold-change in ATAC-seq signal at promoters and enhancers following IFNγ stimulation. **F** Acetylation of H3K27 by ChIP-seq at IFNγ-induced loci. **G** Genes associated with super-enhancers in IFNγ stimulated or control AT3 cells. **H** Core CRC in IFNγ-stimulated cells (new IFNγ-dependent nodes colored red). **I** Binding of BRD4 by ChIP-seq to IFNγ-induced loci. **J** Binding of RNAPII by ChIP-seq to IFNγ-induced loci. **K** Tri-methylation of H3K4me3 by ChIP-seq at IFNγ-induced loci. **L** Histone modifications and RNAPII occupancy at constitutively expressed, IFNγ-induced, or non-expressed loci in AT3 cells. **M** Model for epigenetic activation of IFNγ stimulated genes. ****p < 0.0001, Mann–Whitney *U* test

Based on the prevalence of IRF motifs, we performed ChIP-seq for Irf1 following treatment with IFNγ, which identified the loci where Irf1 was recruited following IFNγ-stimulation (Fig. 2A). As shown in Additional file 1: Fig. S1D, these elements were predominantly localized at intergenic enhancers. It has been reported that IRF TFs physically associate with lysine acetyltransferase (KAT) P300 to activate gene expression [14]]. Therefore, we performed ChIP-seq for P300, which revealed robust co-recruitment to Irf1-bound *cis*-regulatory elements following IFNγ-stimulation (Fig. 2B). Assay for Transposase-Accessible Chromatin using sequencing (ATAC-seq) analyses revealed that under steady-state conditions, Irf1-associated *cis*-regulatory elements exhibit low chromatin accessibility, which was drastically increased following acute IFNγ stimulation (Fig. 2C). Indeed, direct correlation of changes in Irf1 and P300 binding with those regions exhibiting increased chromatin accessibility demonstrated a clear association (Fig. 2D). Moreover, enhancer regions exhibited the greater increases in chromatin accessibility relative to promoters regions (Fig. 2E). Finally, to validate these Irf1 peak-centric analyses, de novo motif analysis of ATAC-seq peaks that were gained in IFNγ-stimulated cells revealed that IRF motifs are significantly overrepresented (Additional file 1: Fig. S1E). Overall, these data demonstrate that IFNγ stimulation leads to rapid co-recruitment of Irf1 and P300 at selective loci, de novo chromatin remodeling, increased chromatin accessibility and RNAPII recruitment.

### IFNγ promotes differential usage of pro-inflammatory enhancers

To test whether recruitment of P300 would lead to hyper-acetylation of adjacent nucleosomes, ChIP-seq for H3K27 acetylation (H3K27ac) was performed. Nucleosomes flanking Irf1-bound sites exhibited greatly increased H3K27ac following IFNγ stimulation (Fig. 2F), directly linking Irf1-P300 co-recruitment with local changes in histone acetylation and chromatin accessibility. To investigate changes in H3K27ac more broadly, and infer differential usage of specific enhancers, we identified super-enhancers (SEs) based on H3K27ac signals. Following IFNγ stimulation, there was acquisition of new SE elements proximal to genes involved in IFNγ signaling (Fig. 2G). These data indicate that IFNγ stimulation leads to acute histone acetylation which correlates with an active transcriptional state at specific enhancer elements adjacent to key pro-inflammatory TFs.

To interrogate differential TF usage, ATAC-seq data were further explored to identify TF motifs overrepresented within putative SEs [15]. These autoregulatory sets of TFs, termed the ‘core regulatory circuit’ (CRC), are represented by calculating the number of individual TF motifs within an SE element regulating the expression of each CRC TF (inward binding) and the number of TF-associated SEs bound by a CRC TF (outward binding). Prior to IFNγ stimulation, the TFs demonstrating the highest connectivity were dominated by TFs previously identified for regulating facets of breast cancer biology (Fig. 2H; in black), including SOX9, GATA3, and ETV6 [16–18]. However, IFNγ stimulation dynamically altered the CRC TF connectivity and led to the acquisition of several interconnected TF nodes involved with pro-inflammatory gene expression (Fig. 2H), including IRF1, IRF2, STAT1, NFKB1, and BCL6. These findings were also recapitulated by calculating the clique fraction, a metric for the participation of individual TFs within interconnected TF networks as a function of the total number of discrete networks (see methods) (Additional file 1: Fig. S1F-G). Thus, IFNγ acutely alters the central repertoire of interconnected TFs that cooperatively regulate cellular transcription.

The Bromodomain and Extra-Terminal (BET) proteins bind acetylated histones and TFs and recruit pTEF-b, thereby stimulating transcriptional elongation [19, 20]. Analysis of BRD4 ChIP-seq data showed robust recruitment of BRD4 following IFNγ stimulation to Irf1-bound loci (Fig. 2I), which may drive effective pause-release post RNAPII initiation (Fig. 1E). Finally, ChIP-seq analysis of RNAPII and H3K4 tri-methylation (H3K4me3), a histone mark that is constitutively associated with active TSS regions (independent of IFNγ stimulation), revealed that RNAPII recruitment in response to IFNγ occurred at promoter-proximal regions demarcated by H3K4me3 (Fig. 2J-K). Overall, these data provide a putative epigenetic sequence of events whereby recruitment of IRF1 in association with P300 leads to locus-specific chromatin remodeling and histone acetylation, a mark subsequently recognized by “reader” proteins, such as BRD4, RNAPII recruitment and transcriptional activation of IFNγ target genes, which are largely inactive under baseline conditions (Fig. 2L-M).

### Inhibition of BET proteins selectively disrupts IFNγ target gene expression

**Fig. 3:**
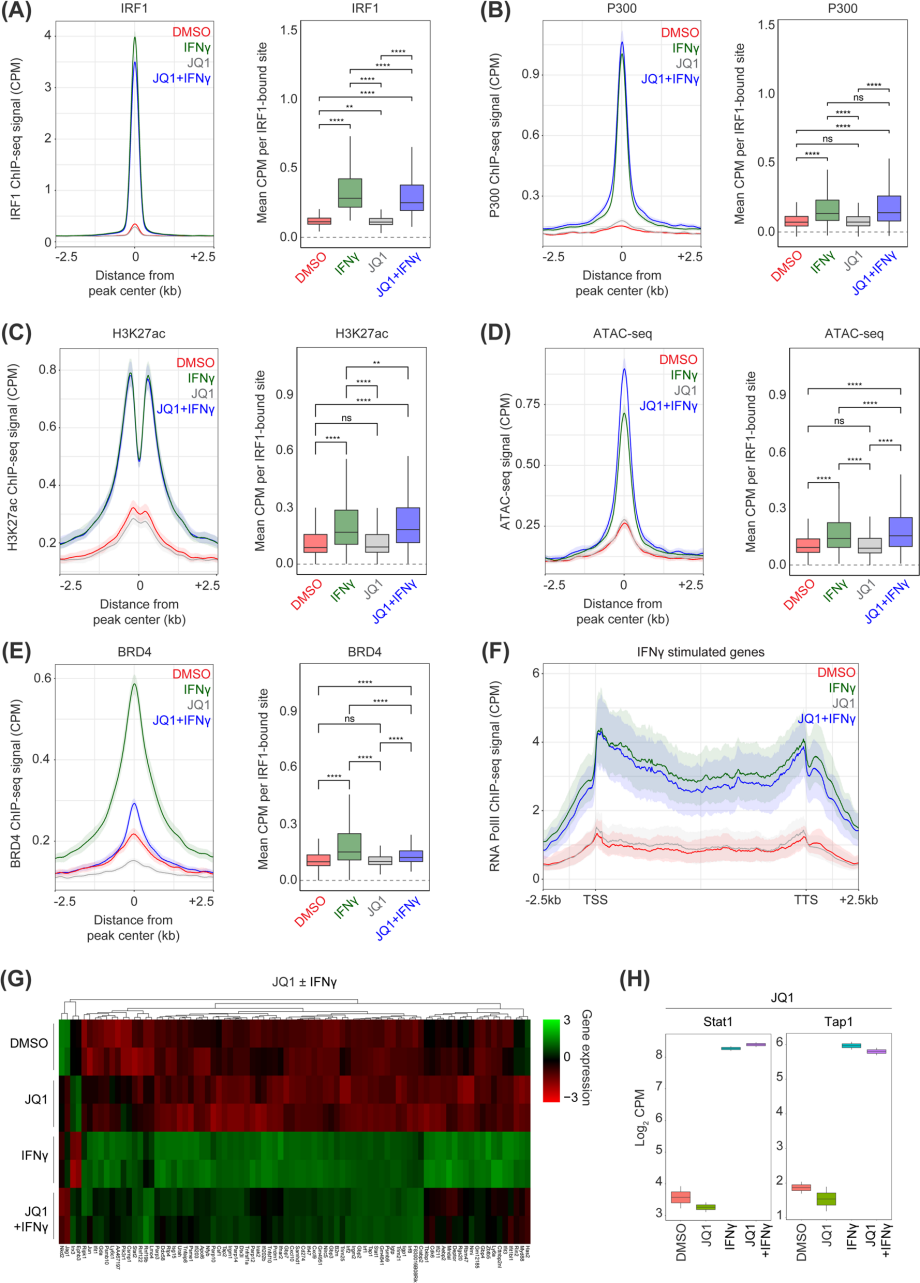
BET inhibition disrupts BRD4 and RNA Pol II recruitment downstream of nucleosome acetylation. **A** Binding of IRF1 by ChIP-seq to IFNγ-induced loci following IFNγ ± JQ1. **B** Binding of P300 by ChIP-seq to IFNγ-induced loci following IFNγ ± JQ1. **C** Chromatin accessibility by ATAC-seq at IFNγ-induced loci following IFNγ ± JQ1. **D** Acetylation of H3K27 by ChIP-seq at IFNγ-induced loci following IFNγ ± JQ1. **E** Binding of BRD4 by ChIP-seq to IFNγ-induced loci following IFNγ ± JQ1. **F** RNA polymerase II occupancy by ChIP-seq across IFNγ stimulated genes following IFNγ ± JQ1. **G** Column-normalized heatmap of gene expression by RNA-seq for IFNγ stimulated genes in the presence of IFNγ ± JQ1. **H** Normalized counts (log_2_ counts per million) of *Stat1* and *Tap1* following IFNγ ± JQ1. *p < 0.05; **p < 0.01; ***p < 0.001; ****p < 0.0001, Mann–Whitney *U* test

To experimentally dissect our epigenetic model of IFNγ-induced gene expression, BET proteins were antagonized using JQ1 to assess where in the cascade BET proteins were required. According to our sequential model, abrogating BET protein recruitment through JQ1 should interfere with IFNγ-driven transcription, but leave the epigenetic remodeling upstream of nucleosome acetylation unperturbed. Consistent with this hypothesis, Irf1 (Fig. 3A) and P300 (Fig. 3B) recruitment, as well as H3K27 acetylation (Fig. 3C), were not significantly affected by BET protein inhibition. Similarly, ATAC-seq revealed that the de novo chromatin remodeling was not reduced by JQ1 co-treatment (Fig. 3D). In contrast, JQ1 co-treatment with IFNγ impaired the recruitment of BRD4 (Fig. 3E) and led to a modest reduction in RNAPII processivity across IFNγ-induced genes (Fig. 3F). Finally, to evaluate the consequences of BET inhibition on mRNA production, we performed RNA-seq under the same conditions. Here, JQ1 co-treatment was associated with highly selective disruption of IFNγ target gene expression, whereby one subset of IFNγ-induced genes was potently suppressed (Fig. 3G), whereas another subset was unaffected by JQ1 co-treatment and remained highly expressed. For example, certain IFNγ-stimulated genes, such as *Stat1* and *Tap1*, were impervious to JQ1 co-treatment (Fig. 3H), whereas immune-suppressive PD-L1 expression was potently suppressed by JQ1-treatment (Fig. 3G). This transcriptional dichotomy remains poorly understood, but suggests that for a subset of genes, effective pause-release can be mediated independently of BRD4 histone-acetyl binding, which is consistent with the notion that the Super Elongation Complex and P-TEFb (CDK9 and Cyclin T) can be recruited in a BRD4 independent manner [21]. These findings are also consistent with numerous studies demonstrating that sub-classification by SEs or other epigenetic/transcriptional features remains insufficient for accurately predicting sensitivity to BET inhibition [22]. Under baseline conditions, JQ1 treatment globally reduced BRD4 binding to chromatin (Additional file 2: Fig. S2A) without altering global chromatin accessibility (Additional file 2: Fig. S2B), even at those regions exhibiting the most robust loss of BRD4 (Additional file 2: Fig. S2C-D), consistent with a recent report [23]. Overall, BET inhibitors reduce BRD4 binding to chromatin and can suppress gene expression downstream of IRF1-driven de novo enhancer remodeling only at a subset of genes/loci.

### Contrasting BET Bromodomain inhibition and P300/CBP acetyltransferase inhibition upon steady-state and IFNγ-induced gene expression

**Fig. 4:**
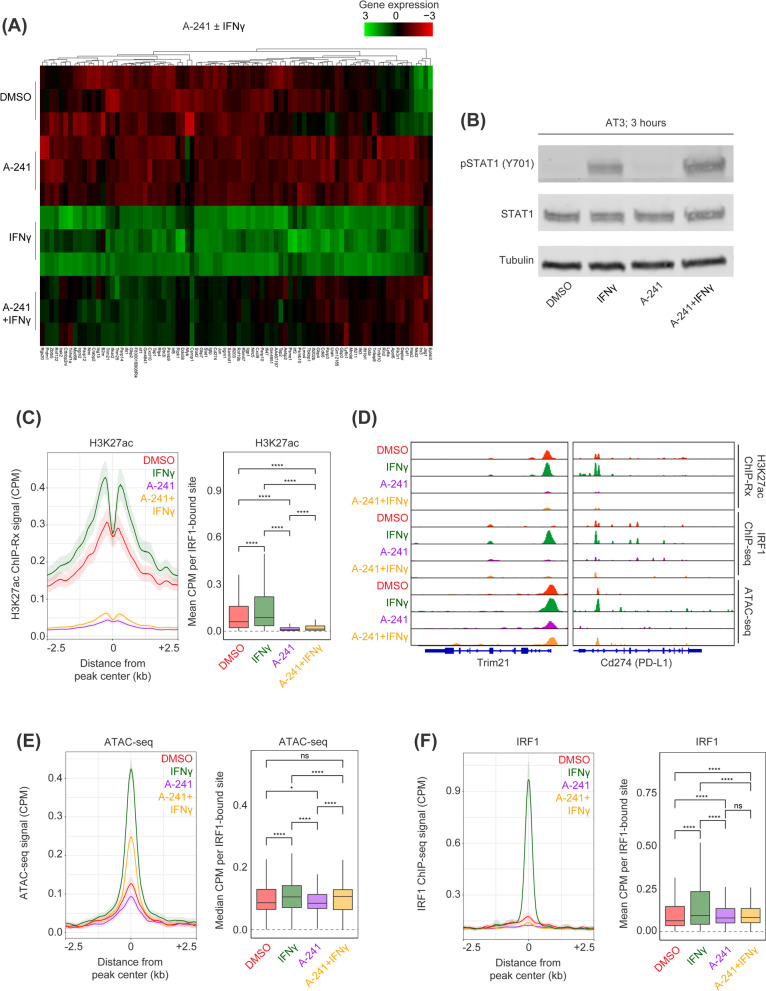
Catalytic P300/CBP inhibition stymies IRF1-dependent transactivation of IFNγ-induced genes. **A** Column-normalized heatmap of gene expression by RNA-seq for IFNγ stimulated genes in the presence of IFNγ ± A-241. **B** AT3 cells stimulated with IFNγ ± A-241 for 3 h prior to immunoblot for Tubulin, STAT1, or phosphor-STAT1 (Y701). **C** Acetylation of H3K27 by ChIP-seq at IFNγ-induced loci following IFNγ ± A-241. **D** IGV screenshot of *Trim21*, *Igtp*, and *Cd274* loci showing H3K27ac ChIP-Rx, ATAC-seq, and IRF1 ChIP-seq in the presence of IFNγ ± A-241. **E** Binding of IRF1 by ChIP-seq to IFNγ-induced loci following IFNγ ± A-241. **F** Chromatin accessibility by ATAC-seq at IFNγ-induced loci following IFNγ ± A-241. *p < 0.05; **p < 0.01; ***p < 0.001; ****p < 0.0001, Mann–Whitney *U* test

Next, the effect of BET protein inhibition was compared with the effects of inhibiting the catalytic KAT domain of lysine acetyltransferase paralogues, P300 and CBP. We assessed the transcriptional consequences on both steady-state and IFNγ-inducible gene expression using two chemically distinct catalytic P300/CBP inhibitors, A-485 [24] and A-241 [25]. RNA-seq under steady-state transcription demonstrated that both A-485 and A-241 preferentially suppressed transcription (Additional file 3: Fig. S3A), although A-241 was more potent (Additional file 3: Fig. S3B, C). Accordingly, A-241 was utilized for all subsequent assays. We performed ChIP-seq with reference exogenous genome (ChIP-Rx) for H3K27ac as a direct biomarker of P300/CBP activity following A-241 treatment. Indeed, H3K27ac signal at active *cis*-regulatory elements demonstrated drastic global reduction following treatment with A-241 (Additional file 3: Fig. S3D) that was not clearly associated with modulation in chromatin accessibility by ATAC-seq (Additional file 3: Fig. S3E, F). Finally, it was notable that the transcriptional response to A-241 and JQ1 was highly divergent (Additional file 4: Fig. S4A), supporting observations recently made in multiple myeloma [26]. The effects of inhibiting P300/CBP catalytic KAT activity on IFNγ-induced gene expression were explored next. Recapitulating the effects on steady-state transcription (Additional file 3: Fig. S3B, C), A-241 more potently suppressed IFNγ-induced gene expression than A-485 (Additional file 4: Fig. S4B). It was clear that while BET inhibition selectively disrupted the transcriptional response to IFNγ (Fig. 3G), the effects of inhibiting P300/CBP catalytic KAT activity were more global (Fig. 4A) and included transcripts unaffected by BET inhibition, such as *Stat1* and *Tap1* (Additional file 4: Fig. S4C). Despite suppressing Stat1 transcriptional up-regulation (Additional file 4: Fig. S4B), A-241 had no effect on phosphorylation of Stat1 (Y701) downstream of IFNγ receptor stimulation (Fig. 4B), highlighting that inhibition of P300/CBP catalytic KAT activity acts downstream of initial signal transduction. Moreover, global analysis of IFNγ-stimulated genes demonstrated more robust suppression when compared to BET inhibition (Additional file 4: Fig. S4D). These findings highlight that inhibiting P300/CBP catalytic KAT activity robustly modulates cellular transcription. Importantly, these transcriptional defects are broader and clearly dissimilar to the more selective effects of BET bromodomain inhibition, which we anticipate would be even more pronounced if nascent RNA profiling was employed.

### Loss of P300/CBP lysine acetyltransferase activity prevents activation of IRF1-bound cis-regulatory elements

To determine how inhibition of P300/CBP impacted the epigenetic remodeling induced by IFNγ, ChIP-Rx [27] for H3K27ac was utilized to investigate the capacity for A-241 to suppress hyper-acetylation of IRF1-associated *cis*-regulatory elements. As expected, A-241 co-treatment was able to potently suppress IFNγ-induced histone hyper-acetylation (Fig. 4C, D). The requirement of lysine acetylation by P300/CBP for chromatin accessibility during a de novo remodeling process and transcriptional activation process was unclear. Therefore, ATAC-seq in the presence and absence of IFNγ and A-241 was performed, assessing the changes in chromatin accessibility at IRF1-associated *cis*-regulatory elements. Catalytic P300/CBP KAT activity was required, at least in part, for chromatin accessibility changes associated with IFNγ stimulation (Fig. 4D-E). ChIP-seq for Irf1 under those conditions was also performed, given that Irf1 binding was a critical prerequisite to epigenetic activation of these loci. Strikingly, we found that inhibiting P300/CBP catalytic KAT activity almost completely inhibited the transactivation by Irf1 (Fig. 4D, F), thereby linking loss of TF binding with a failure to subsequently gain chromatin accessibility. Overall, these data indicate that acetylation by P300/CBP regulates transactivation by IRF1 following IFNγ stimulation. More broadly, these findings highlight that P300/CBP are a critical epigenetic dependency that underpins the transcriptional response to IFNγ.

## Discussion

Inhibiting the lysine acetyltransferase activity of P300/CBP is an emergent therapeutic strategy in cancer. First-generation catalytic P300/CBP inhibitor, A-485, was shown to possess anti-tumor activity in models of prostate cancer [24], multiple myeloma (MM), and chronic lymphocytic leukemia [26]. Using a second-generation P300/CBP inhibitor A-241, we could recapitulate key recent findings that showed acute maintenance of chromatin accessibility occurred largely independently of histone acetylation by P300/CBP [26, 28, 29]. This may be, at least in part, due to P300 remaining bound to chromatin, while its catalytic KAT domain is inhibited, as was shown in MM [26]. One possibility is that combining P300/CBP catalytic KAT inhibitors with P300/CBP Bromodomain inhibitors (to promote displacement of P300/CBP from chromatin) could more profoundly disrupt P300/CBP protein complexes, leading to more pronounced disruption of epigenetic and transcriptional processes. In agreement with this notion, combined inhibition of P300/CBP KAT and Bromodomain modules in MM was shown to additively promote histone hypoacetylation, though these effects were less pronounced than a dual P300/CBP-targeting proteolysis-targeting chimera (PROTAC) molecule, dCBP-1, which additionally reduced chromatin accessibility [42]. Indeed, emergent small molecule inhibitors and PROTACs targeting P300 and/or CBP will allow for the systematic characterization of the additional functions of P300/CBP beyond its KAT activity, such as the known scaffolding functionality.

In contrast to the effects of inhibiting P300/CBP catalytic KAT activity upon cells under steady-state conditions, chromatin accessibility associated with an acute transcriptional stimulus such as IFNγ was highly dependent on the catalytic KAT activity of P300/CBP. While inhibiting P300/CBP catalytic KAT activity abrogated IFNγ-induced expression of certain antigen processing genes (e.g. Tap1), which may potentially limit CD8^+^ T-cell immunity, this may concomitantly augment natural killer cell-driven killing. Conversely, it is plausible that catalytic P300/CBP inhibitors may be leveraged in auto-immune disorders and graft-vs-host disease to reduce unwanted or excessive T-cell mediated immune responses. Thus, the overall net effect of therapeutically inhibiting P300/CBP catalytic KAT activity on immune responses remains unclear and requires pre-clinical evaluation using syngeneic in vivo model systems. These in vivo studies will be essential to provide independent validation of the epigenetic mechanisms demonstrated here, as well as investigation of the effects of inhibiting P300/CBP catalytic KAT activity on the host immune system and anti-tumor immunity.

We demonstrated that catalytic P300/CBP KAT activity is required for IRF1 transactivation following IFNγ stimulation. We note this effect could be potentially resultant from (1) P300/CBP suppressing transcription of IRF1 itself, (2) loss of a functional acetylation site on IRF1, (3) P300/CBP inhibitors antagonizing the acetylation-independent allosteric interaction between IRF1 and P300 [30], or (4) a combination of (1)–(3). Thus, systematic biochemical assays will be required to deconvolute these possibilities, as well as the broader conservation of these mechanisms across distinct cell/tumor types.

## Conclusions

This study provides an epigenetic hierarchy for IFNγ-stimulated gene expression. Previous efforts to clarify dependencies of IFNγ target genes have largely been performed using genetic depletion approaches, which are particularly problematic for studying the epigenetic proteins that are typically pan-essential. Moreover, these studies have historically been limited to a single IFNγ responsive locus, which fails to capture the global complexity. In contrast, the work detailed herein utilized integrated and unbiased genomics methodologies to evaluate the hierarchy of events that leads to induction of transcription following IFNγ stimulation, as well as functional studies to evaluate this model by antagonizing various epigenetic regulators. Overall, these findings provide fundamentally important insight into the presumed role for certain epigenetic regulators in driving expression of IFNγ target genes, while also highlighting the importance of P300/CBP KAT activity for IRF1 transactivation.

## Methods

### Cell lines and reagents

AT3 breast cancer cell lines were obtained from the institutional cell line bank at the Peter MacCallum Cancer Center (PMCC; Melbourne, Australia) and tested bi-monthly for the presence of mycoplasma. Cells were cultured in vitro for < 3 months before a fresh aliquot was thawed to minimize culture adaptations/genetic drift. AT3 cells were grown at 37 °C and 10% CO_2_ in Gibco Dulbecco's Modified Eagle Medium (DMEM) supplemented with 10% fetal calf serum, penicillin (100 u/mL), and streptomycin (100 mg/mL). JQ1 was provided by Dr. James E. Bradner (Boston MA, USA). Catalytic P300/CBP inhibitors A-241 and A-485 were provided by AbbVie (North Chicago, IL, USA). All small molecules were reconstituted at 10 mM in 100% dimethyl-sulfoxide (DMSO) and stored at – 20 °C. Recombinant murine and human IFNy was purchased from BioLegend (Catalog #575304 and # 570206, respectively) and diluted to 20 µg/mL in 0.5% BSA in PBS and stored in single-use aliquots at – 80 °C.

### Immunoblot

AT3 cells were pre-treated with A-241 (250 nM) for 1 h prior to the addition of recombinant murine IFNy (1 ng/mL) for an additional 2 h. Cells were harvested by centrifugation and washed once in ice-cold PBS prior to whole cell lysis using Lamelli buffer (60 mM Tris HCl pH 6.8, 10% v/v glycerol, 2% v/v glycerol SDS) and incubated at 95 °C for 5–10 min until completely homogenized. Cell lysate protein concentration was measured using Pierce BCA Protein Assay Kit (ThermoFisher Scientific, 23225) according to the manufacturer’s instructions. Prior to running SDS-PAGE, protein lysates were prepared with sample loading buffer (120 mM Tris HCl pH 6.8, 20% v/v glycerol, 4% w/v SDS, 71.5 mM β-mercaptoethanol, bromophenol blue). Protein lysates were separated on Mini-PROTEAN TGX 4–15% gels (Bio-Rad, 465-1086) prior to transfer at 0.25A onto Immobilon-P (IPVH00010) membranes in transfer buffer (25 mM Tris HCl, 192 mM Glycine, 5% v/v methanol) at 4 °C. Membranes were blocked with Tris-buffered saline (TBS) supplemented with 5% w/v skim milk powder. Next, membranes were incubated overnight using the following primary antibodies: anti-phospho[Y701]-STAT1 (#9167, Cell Signaling Technologies), anti-STAT1[total] (#610185, BD Biosciences), and anti-α-Tubulin (#05-829, Millipore Sigma). Membranes were incubated with horse radish peroxidase (HRP)-conjugated secondary antibodies at room temperature for 1 h and washed at least three times in TBS supplemented with 0.1% v/v Tween20. Immunoreactive bands were revealed using ECL reagents (Amersham ECL or ECL Prime, GE Healthcare) by film exposure (Fujifilm Super RX, Fujifilm) using an Agfa CP1000 developer (Agfa). For both of the cropped immunoblots presented in Fig. 4B, the corresponding uncropped blots are also shown in Additional File 5: Fig. S5A, B.

### TCGA correlation analysis

RNA-Seq by Expectation Maximization (RSEM) [31] scaled expression values for TCGA were downloaded from the GDAC Firehose website [32]. Entrez gene IDs were mapped to HGNC gene symbols using the biomaRt (v2.42) R package [33] and collapsed to unique values per gene symbol by selecting the most variable entrez ID among all samples for each gene symbol. Primary samples from the TCGA BRCA cohort were selected using the TCGAbiolinks (v2.14.0) R package [34] and were matched with progression-free interval end points from the TCGA Pan-Cancer Clinical Data Resource [35]. IFNg signature scores were calculated using the Singscore (v1.6) R package [36] from a set of genes found to be strongly interferon induced across multiple cell lines (Additional file 1: Fig. S1C). Samples were then stratified into 'High' (top 90th percentile) and 'Low' (bottom 10th percentile) signature score groups and log-rank *p* values were calculated using the Survival (v2.38) R package [37].

### RNA-sequencing

5e6 AT3 cells were plated in technical triplicate and each pre-treated with A-241 (250 nM), A-485 (1 µM), or DMSO vehicle for 1 h prior to the addition of recombinant murine IFNy (1 ng/mL), or vehicle control, for an additional 2 h (3 h total incubation with small molecules). Following indicated treatments, cells were collected by centrifugation and washed once with ice-cold PBS prior to resuspension in TRIzol™ (ThermoFisher Scientific, 15596026). RNA was isolated using the Direct-zol RNA MiniPrep kit (Zymo Research, R2052) according to the manufacturers instructions and eluted in 50 µL nuclease-free H_2_O. Sequencing libraries were prepared by the Molecular Genomics Core Facility (Peter MacCallum Cancer Center) with 500 ng input RNA using the QuantSeq 3’-mRNA Seq Library Prep Kit for Illumina (Lexogen, Vienna, Austria). Libraries were then pooled and sequenced on the Illumina NextSeq 500 to obtain 75 b.p. single-end reads. Sequencing files were demultiplexed using Bcl2fastq (v2.17.1.14) to generate individual FASTQ files on which QC was performed using FASTQC (v0.11.5). Sequencing reads were trimmed using cutadapt (v1.7) and aligned to the mouse reference genome (GRCm38/Mm10) using HISAT2 (v2.1.0). Read counting across genomic features was performed using featureCounts and the following settings: -p -T 20 -O -F GTF -t exon. Differential gene expression analysis was performed on the resultant counts matrix in Rstudio (v3.5.1) using the Limma/Voom workflow [38, 39]. Gene set enrichment analyses were performed using Gene Set Enrichment Analysis (GSEA) software (v3.0; https://www.gsea-msigdb.org/gsea/index.jsp) using pre-ranked (ranked by t-statistic) and enrichment plots were re-plotted from GSEA output using replotGSEA function in Rstudio. All additional figure generation for RNA-sequencing datasets was performed in in Rstudio (v3.5.1).

### ChIP-sequencing

Cells were pre-treated with JQ1 (1 µM), A-241 (250 nM), or A-485 (1 µM) for 1 h prior to the addition of recombinant murine IFNy, or vehicle control, for an additional 2 h (3 h total incubation with small molecules). Chromatin immunoprecipitation coupled with next-generation sequencing (ChIP-seq) was performed with reference exogenous genome (ChIP-Rx) using a modified protocol [27]. MM1.S cells (25e6/IP) were cultured in the presence or absence of A-485 or DMSO vehicle for indicated timepoints. At harvest, cells were washed once in ice-cold PBS prior to cross-linking. For cross-linking, 1/10th volume of fresh formaldehyde solution (11% formaldehyde, 0.05 mM EGTA, 1 mM EDTA, 100 mM NaCl, 50 mM Hepes–KOH pH 7.5) was added and incubated for 20 min at room temperature with rotation. Cross-linking was quenched by the addition of 1/20th volume of 2.5 M glycine and incubated for 5 min at room temperature with rotation. For isolation of nuclei, cell pellets were washed once in ice-cold PBS and then resuspended in ice-cold nuclear extraction buffer (0.5% NP-40, 2 mM EDTA, 10 mM NaCl, 20 mM Tris–HCl pH 8) and incubated for 5 min on ice. Following three sequential incubations in nuclear extraction buffer, cell nuclei were pelleted and resuspended in sonication buffer (0.3% SDS, 1% NP-40, 2 mM EDTA, 150 mM NaCl, 20 mM Tris–HCl pH 7.5) at a concentration equivalent to 50e6 cells per mL. Samples were sonicated in 12 × 24 mm Covaris tubes using the Covaris S2 instrument for 18 min using the following settings: 20% Duty Cycle, 1000 cycles/burst, and 10 Intensity. Prior to immunoprecipitation, sheared chromatin was diluted 1:1 in ChIP dilution buffer (1% Triton X-100, 2 mM EDTA, 150 mM NaCl, 20 mM Tris–HCl pH 8) and quantified using Qubit dsDNA HS assay kit. For ChIP-Rx, sheared Drosophila chromatin from S2 cells was spiked into immunoprecipitations at 1:40 ratio of Drosophila/human and processed as a single sample until ChIP-Rx normalization following DNA sequencing. Immunoprecipitations were performed overnight (12–16 h, 4 °C, with rotation) using Protein A and Protein G Dyna beads (Invitrogen) and the following antibodies: H3K27Ac (Abcam, ab4729) and IRF1 (Santa Cruz Biotechnology Inc., sc-497). Samples were washed once with ChIP dilution buffer, wash buffer 1 (0.1% SDS, 1% Triton X-100, 2 mM EDTA, 500 mM NaCl, 20 mM Tris–HCl pH 8), wash buffer 2 (0.5% deoxycholate, 0.5% NP-40, 2 mM EDTA, 250 mM LiCl, 20 mM Tris–HCl pH 8), and TE buffer (1 mM EDTA, 10 mM Tris–HCl pH 7.5) prior to incubation in reverse cross-linking buffer (200 mM NaCl, 100 mM NaHCO_3_, 1% SDS, 300 μg/mL Proteinase-K) for 4 h at 55 °C with shaking. Finally, the supernatant was reverse-cross-linked overnight (12–16 h) at 65 °C prior to ChIP DNA isolation using Zymogen ChIP DNA Clean and Concentrator Kit (Zymo Research, D5205). For ChIP-Rx, libraries were generated using the NEBNext Ultra II DNA Library Prep Kit (NEB, E7645) and sequenced on an Illumina NextSeq 550 with 75 b.p. single-end reads. Library QC and quantification were performed using D1000 high-sensitivity screen tape with 4200 TapeStation Instrument (Agilent Technologies), and the size is selected between 200 and 500 bp using a Pippin Prep system (Sage Science).

### ATAC-sequencing

Cells were pre-treated with JQ1 (1 µM) or A-241 (250 nM) for 1 h prior to the addition of recombinant murine IFNy (1 ng/mL), or vehicle control, for an additional 2 h (3 h total incubation with small molecules). Assay for Transposase-Accessible Chromatin using Sequencing (ATAC-seq) was performed using an improved protocol to reduce mitochondria from the transposition reaction [40]]. Briefly, 5e5 MM1.S cells were cultured in duplicate with JQ1, A-485, or DMSO vehicle as described above. Cells were washed once in ice-cold PBS and lysed in ATAC lysis buffer (0.1% Tween-20, 0.1% NP-40, 3 mM MgCl_2_, 10 mM NaCl, 10 mM Tris HCl pH 7.4). Tagmentation was then performed with Tn5 transposase and 2 × TD Buffer (Nextera DNA Library Prep Kit, Illumina) for 30 min at 37 °C (in a thermocycler). Tagmented DNA was immediately purified using a MinElute column (Qiagen, #28004) and then amplified for 12 cycles using 2 × KAPA HiFi HotStart ReadyMix (Kapa Biosystems, KK2602) and Illumina-compatible/barcoded primers. The amplified libraries were purified using MinElute columns (Qiagen) and sequenced on an Illumina NextSeq 500 with 75 b.p. single-end reads. Library QC and quantification were performed using D1000 high-sensitivity screen tape with 4200 TapeStation Instrument (Agilent Technologies), and the size was selected between 200 and 500 bp using a Pippin Prep system (Sage Science).

### ATAC-seq and ChIP-seq analysis

Sequencing files were demultiplexed using Bcl2fastq (v2.17.1.14) to generate Fastq files on which QC was performed using FASTQC (v0.11.5). Sequencing reads were then aligned to custom reference genome consisting the mouse genome (Mm10) and the Drosophila melanogaster genome (Dm3) using Bowtie2 (v2.3.3). The resulting SAM files were converted to BAM files using Samtools (v1.4.1) using the view command, which were subsequently sorted, indexed, and potential PCR duplicates removed using the rmdup function. BAM files were converted into BigWig files using the bamCoverage function (Deeptools, v3.0.0) using the following settings (—normalizeUsing CPM—smoothLength 150—binSize 50—e 200 scaleFactor 1). For experiments with external normalization, the reads mapping to either Mm10 or Dm3 genomes were quantified using FeatureCounts (Subread package, v1.5.0) and the percentage of mapped Dm3 reads as a total of total mapped Dm3 + Hg19 reads was calculated. A scale factor was then calculated as the ratio of Dm3 reads in the control treatment condition and the treatment sample, which was then manually applied as the scaleFactor in the bamCoverage function. BigWig files were imported into Integrative Genomics Viewer (IGV, v2.7.0) for visualization of specific loci. Using Deeptools (v3.0.0), heatmaps were generated by computing read average read density (from BigWig files) across defined genomic intervals using computeMatrix, which we subsequently plot using the plotHeatmap command. Average profile plots were created using matrices generated by computeMatrix using a custom script in Rstudio. Annotation of putative super-enhancer regions from H3K27ac ChIP-seq data was performed using Ranking Ordering of Super-Enhancer (ROSE) using a 12.5 k.b. stitching distance and a 2.5 k.b. TSS exclusion to reduce promoter bias. Peak calling was performed with MACS2 with default parameters. Annotation of ATAC-Seq/ChIP-Seq peaks to proximal genes was performed using annotatePeaks.pl (Homer, v4.8). Rstudio (v1.1.46) and R (v3.5.1) were used for all additional analyses and figure preparation using the following R packages: ggplots2, rcolorbrewer.

### Super-enhancer and Coltron analysis

MACS2 (v2.2.1) was used for identification of (1) ATAC-seq peaks on BAM files from vehicle-treated and IFNγ-treated cells using the following parameters:—call-summits—nomodel—extsize 300, and (2) H3K27ac peaks on BAM files from vehicle-treated and IFNγ-treated cells using the following parameters:—cutoff-analysis. ATAC-seq and H3K27ac ChIP-seq peaks (.narrowPeak) mapping to ENCODE’s recommended Blacklisted regions [41] for the Mm10 genome were then excluded using bedtools intersect. Rose2 (v1.0.5) was then used to identify super-enhancers using the following parameters: -g mm10 -s 12,500 -t 2000. Coltron (https://pypi.org/project/coltron/) was used to perform core regulatory circuit analysis using the following parameters: -g MM10 -l 300. Rose2 and Coltron steps were repeated for both vehicle-treated and IFNγ-treated samples. Rstudio (v1.1.46) was used for figure preparation.

### Data availability

RNA-sequencing data of B16-F10 cells stimulated with IFNγ or vehicle control were downloaded from NIH’s Gene Expression Omnibus (GEO) under the accession number GSE134264. RNA-sequencing of MC38 cells stimulated with IFNγ or control was downloaded from GEO (GSE112252). RNA-sequencing of AT3 cells stimulated with IFNγ ± JQ1, or vehicle control, from our previous study was downloaded from GEO (GSE94057). ChIP-sequencing for RNA polymerase II, BRD4, IRF1, and H3K27ac in AT3 cells stimulated with IFNγ ± JQ1, or vehicle control, from our previous study was downloaded from GEO (GSE94130). Next-generation sequencing data generated in this study have been deposited in the NIH’s Gene Expression Omnibus (GEO) under the accession number GSE201883.

## Supplementary Information

**Additional file 1: Fig S1**. Conserved activation of IRF1 in response to IFNγ. **(A)** RNA-seq of B16-F10 melanoma cells stimulated with IFNγ. **(B)** RNA-seq of MC38 colon adenocarcinoma cells stimulated with IFNγ. **(C)** Venn diagram overlap of genes induced by IFNγ stimulation (Log_2_ fold-change > 1, P-value < 0.01) in AT3, B16-F10, and MC38 cells. **(D)** Genomic localization of IRF1-bound *cis*-regulatory elements identified following IFNγ stimulation. **(E)** De novo motif analysis in ATAC-seq peaks specifically found in IFNγ stimulated AT3 cells using peaks found in vehicle-treated AT3 cells as the background. **(F)** Clique fraction for highly connected core transcription factors in vehicle-treated cells. **(G)** Clique fraction for highly connected core transcription factors in IFNγ-treated cells.

**Additional file 2: Fig S2**. BET inhibition with JQ1 depleted BRD4 binding genome-wide. **(A)** BRD4 ChIP-seq signal (normalized log_2_ counts per million) in presence of JQ1 (1 µM) or DMSO for 3 h at cis-regulatory elements active under baseline conditions (in the absence of IFNγ) as identified by ATAC-seq. **(B)** ATAC-seq signal (normalized log_2_ counts per million) at cis-regulatory elements active under baseline conditions (in the absence of IFNγ) as identified by ATAC-seq. **(C)** Log_2_ fold-change in BRD4 signal (by ChIP-seq) at active cis-regulatory elements across 10 quantiles ranked by loss of BRD4. **(D)** Log_2_ fold-change in ATAC-seq signal at active cis-regulatory elements across 10 quantiles ranked by loss of BRD4. ****p < 0.0001, Mann–Whitney U test.

**Additional file 3: Fig S3**. Catalytic P300/CBP inhibitors perturb cellular transcription. **(A)** RNA-seq AT3 cells treated for 3 h with A-485 (1 µM) or A-241 (250 nM) relative to DMSO. **(B)** Venn diagram overlap of differentially expressed genes (P-value < 0.01) following treatment of AT3 cells with A-485 or A-241 relative to DMSO, respectively. **(B)** Row-normalized heatmap of gene expression values from RNA-seq of AT3 cells treated with A-485, A-241, or DMSO vehicle for genes responsive to A-241 treatment (P-value < 0.01). **(D)** H3K27ac ChIP-Rx signal (normalized log_2_ counts per million) in presence of A-241 or DMSO for 3 h at cis-regulatory elements active under baseline conditions (in the absence of IFNγ) as identified by ATAC-seq. **(E)** Log_2_ fold-change in H3K27ac ChIP-Rx at active cis-regulatory elements across 10 quantiles ranked by loss of H3K27ac. **(F)** Log_2_ fold-change in ATAC-seq signal at active cis-regulatory elements across 10 quantiles ranked by loss of H3K27ac. ****p < 0.0001, Mann–Whitney U test.

**Additional file 4: Fig S4**. P300/CBP and BET inhibition have disparate effects on steady-state and IFNγ-inducible transcription. **(A)** Venn diagram overlap of genes down-regulated following 3 h treatment with JQ1 and A-241 (P-value < 0.05, log_2_ fold-change < -1 relative to DMSO) in AT3 cells. **(B)** Row-scaled heatmap of normalized gene expression values for (log_2_ counts per million) of IFNγ-induced genes following treatment of AT3 cells with IFNγ ± A-241 or A-485, respectively. **(C)** Normalized counts (log_2_ counts per million) of *Stat1* and *Tap1* following IFNγ ± A-241. **(D)** Normalized counts (log_2_ counts per million) of IFNγ-induced genes following treatment of AT3 cells with IFNγ ± JQ1 or IFNγ ± A-241. *p < 0.05, ****p < 0.0001 t test.

**Additional file 5: Fig S5**. Uncropped Western blot images. **(A)** Uncropped Western blot image of data presented in Fig. 4B, corresponding to total Stat1 and Tubulin. **(B)** Uncropped Western blot image of data presented in Fig. 4B, corresponding to Phospho-Stat1 [Y701] and Tubulin.

## Data Availability

Next-generation sequencing data generated in this study have been deposited in the NIH’s Gene Expression Omnibus (GEO) under the accession number GSE201883.

## Abbreviations

ATAC-seq: Assay for Transposase-Accessible Chromatin with high-throughput sequencing
BET: Bromodomain and extra-terminal domain
CBP: CREB binding protein
ChIP-seq: Chromatin immunoprecipitation and sequencing
CRC: Core regulatory circuit
GSEA: Gene set enrichment analysis
IFNγ: Interferon gamma
IRF: Interferon-regulatory factors
ISRE: Interferon-sensitive response element
KAT: Lysine acetyltransferase
PROTAC: Proteolysis-targeting chimera
P-TEFb: Positive transcription elongation factor b
RNAPII: RNA Polymerase II
RNA-seq: RNA sequencing
SE: Super-enhancer
TF: Transcription factor
TME: Tumor microenvironment
