# Supplementary figures and images for: Distinct modulation of IFNγ-induced transcription by BET bromodomain and catalytic P300/CBP inhibition in breast cancer
Source: Clin Epigenetics. 2022 Jul 28;14:96. doi: 10.1186/s13148-022-01316-5 (PMC9336046; doi:10.1186/s13148-022-01316-5)

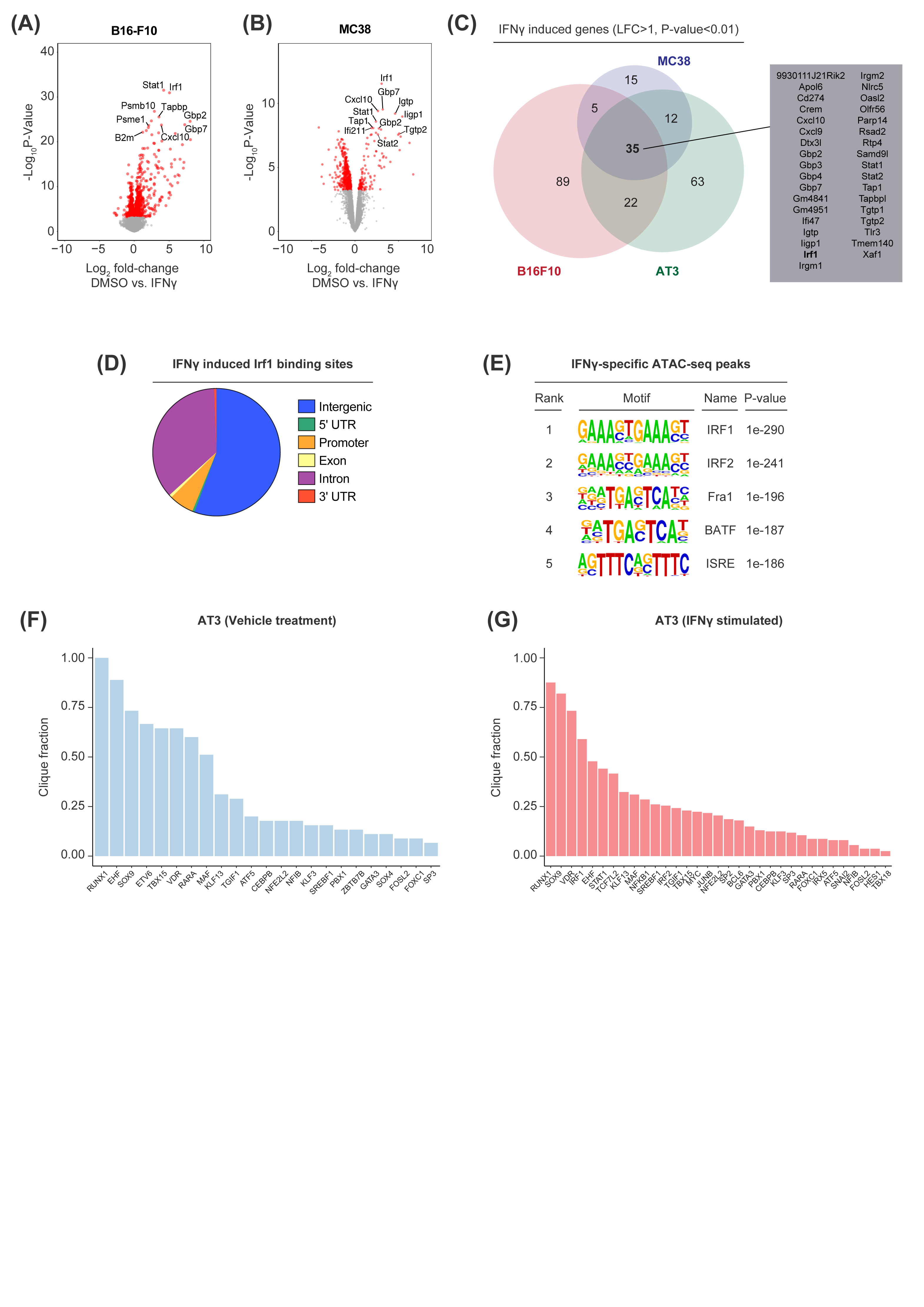

Supplement: Supplementary file 1 — Additional file 1: Fig S1. Conserved activation of IRF1 in response to IFNγ. (A) RNA-seq of B16-F10 melanoma cells stimulated with IFNγ. (B) RNA-seq of MC38 colon adenocarcinoma cells stimulated with IFNγ. (C) Venn diagram overlap of genes induced by IFNγ stimulation (Log2 fold-change > 1, P-value < 0.01) in AT3, B16-F10, and MC38 cells. (D) Genomic localization of IRF1-bound cis-regulatory elements identified following IFNγ stimulation. (E) De novo motif analysis in ATAC-seq peaks specifically found in IFNγ stimulated AT3 cells using peaks found in vehicle-treated AT3 cells as the background. (F) Clique fraction for highly connected core transcription factors in vehicle-treated cells. (G) Clique fraction for highly connected core transcription factors in IFNγ-treated cells. [file 13148_2022_1316_MOESM1_ESM.tif]

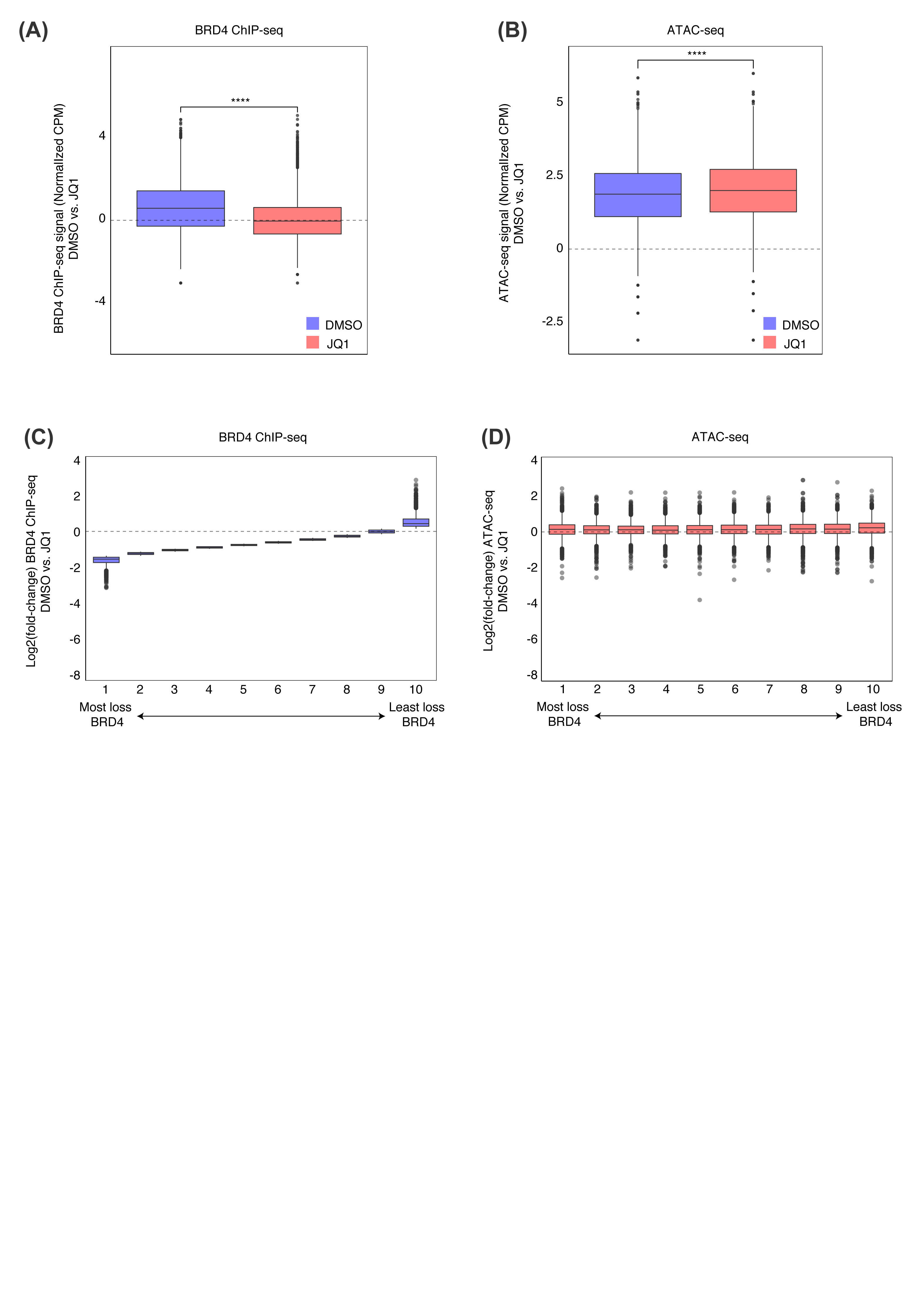

Supplement: Supplementary file 2 — Additional file 2: Fig S2. BET inhibition with JQ1 depleted BRD4 binding genome-wide. (A) BRD4 ChIP-seq signal (normalized log2 counts per million) in presence of JQ1 (1 µM) or DMSO for 3 h at cis-regulatory elements active under baseline conditions (in the absence of IFNγ) as identified by ATAC-seq. (B) ATAC-seq signal (normalized log2 counts per million) at cis-regulatory elements active under baseline conditions (in the absence of IFNγ) as identified by ATAC-seq. (C) Log2 fold-change in BRD4 signal (by ChIP-seq) at active cis-regulatory elements across 10 quantiles ranked by loss of BRD4. (D) Log2 fold-change in ATAC-seq signal at active cis-regulatory elements across 10 quantiles ranked by loss of BRD4. ****p < 0.0001, Mann–Whitney U test. [file 13148_2022_1316_MOESM2_ESM.tif]

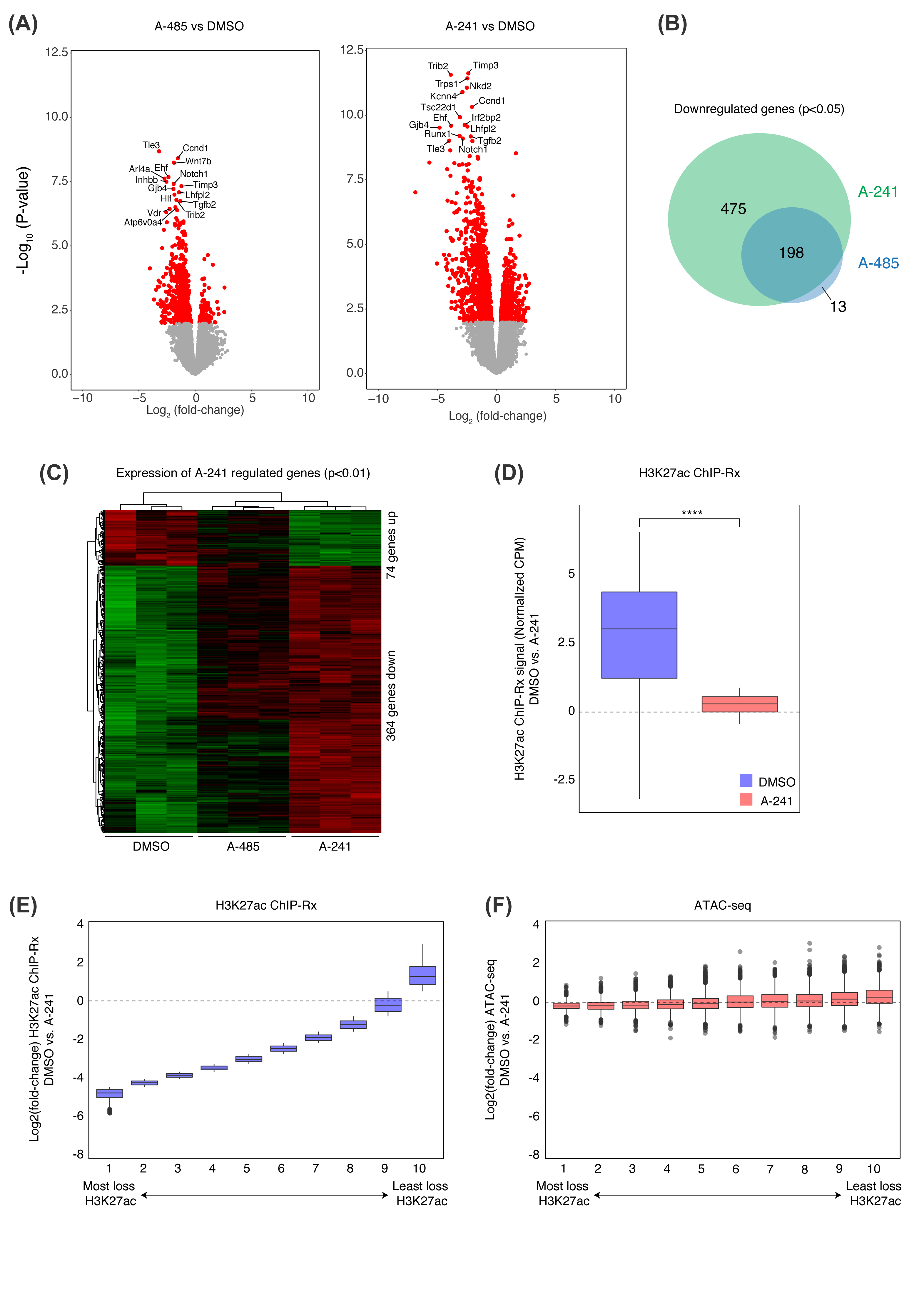

Supplement: Supplementary file 3 — Additional file 3: Fig S3. Catalytic P300/CBP inhibitors perturb cellular transcription. (A) RNA-seq AT3 cells treated for 3 h with A-485 (1 µM) or A-241 (250 nM) relative to DMSO. (B) Venn diagram overlap of differentially expressed genes (P-value < 0.01) following treatment of AT3 cells with A-485 or A-241 relative to DMSO, respectively. (B) Row-normalized heatmap of gene expression values from RNA-seq of AT3 cells treated with A-485, A-241, or DMSO vehicle for genes responsive to A-241 treatment (P-value < 0.01). (D) H3K27ac ChIP-Rx signal (normalized log2 counts per million) in presence of A-241 or DMSO for 3 h at cis-regulatory elements active under baseline conditions (in the absence of IFNγ) as identified by ATAC-seq. (E) Log2 fold-change in H3K27ac ChIP-Rx at active cis-regulatory elements across 10 quantiles ranked by loss of H3K27ac. (F) Log2 fold-change in ATAC-seq signal at active cis-regulatory elements across 10 quantiles ranked by loss of H3K27ac. ****p < 0.0001, Mann–Whitney U test. [file 13148_2022_1316_MOESM3_ESM.tif]

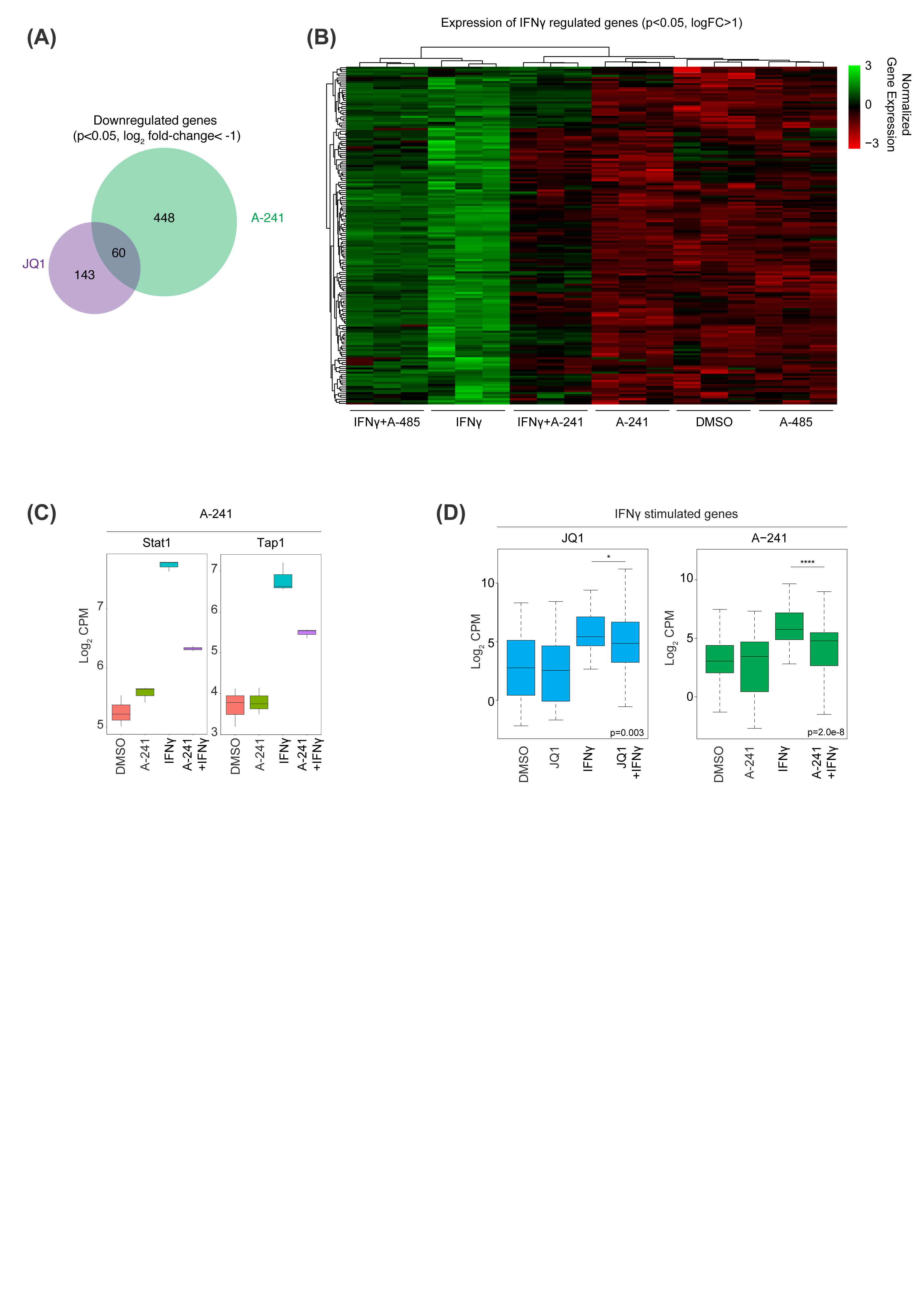

Supplement: Supplementary file 4 — Additional file 4: Fig S4. P300/CBP and BET inhibition have disparate effects on steady-state and IFNγ-inducible transcription. (A) Venn diagram overlap of genes down-regulated following 3 h treatment with JQ1 and A-241 (P-value < 0.05, log2 fold-change < -1 relative to DMSO) in AT3 cells. (B) Row-scaled heatmap of normalized gene expression values for (log2 counts per million) of IFNγ-induced genes following treatment of AT3 cells with IFNγ ± A-241 or A-485, respectively. (C) Normalized counts (log2 counts per million) of Stat1 and Tap1 following IFNγ ± A-241. (D) Normalized counts (log2 counts per million) of IFNγ-induced genes following treatment of AT3 cells with IFNγ ± JQ1 or IFNγ ± A-241. *p < 0.05, ****p < 0.0001 t test. [file 13148_2022_1316_MOESM4_ESM.tif]

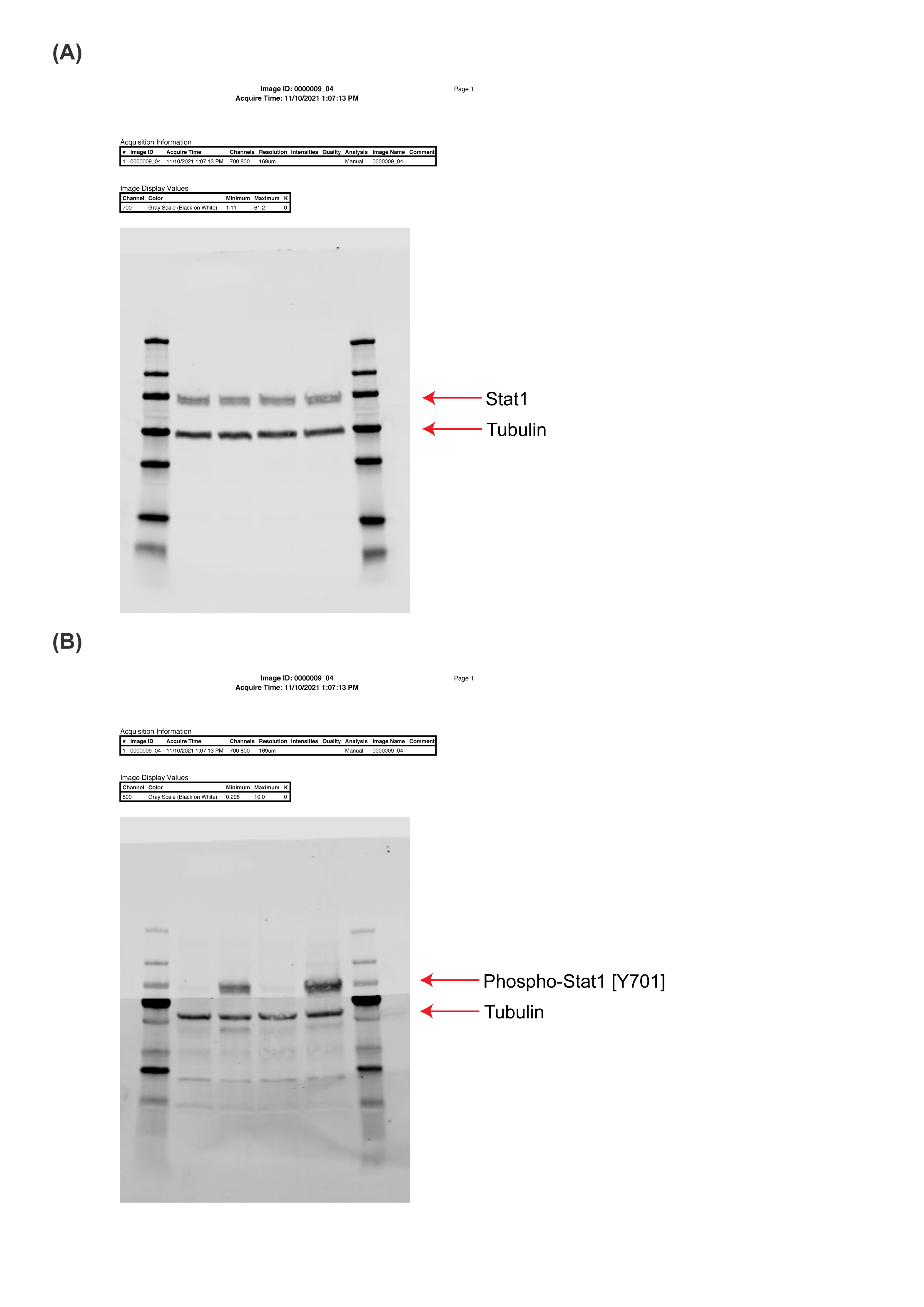

Supplement: Supplementary file 5 — Additional file 5: Fig S5. Uncropped Western blot images. (A) Uncropped Western blot image of data presented in Fig. 4B, corresponding to total Stat1 and Tubulin. (B) Uncropped Western blot image of data presented in Fig. 4B, corresponding to Phospho-Stat1 [Y701] and Tubulin. [file 13148_2022_1316_MOESM5_ESM.tif]
